# 

**Published:** 1890-02

**Authors:** 


### Society Notes.

### TEXAS STATE MEDICAL ASSOCIATION.

OFFICE OF THE EXECUTIVE,
AUSTIN, TEXAS, Feb. 10, 1890.

*To the Members of the Association*—Greeting:

In obedience to the requirements of our by-laws, and conformity to a time-honored custom, it is incumbent on your Executive to remind you that the Twenty-second Annual Convention of the Association will be held in the city of Fort Worth, the fourth week in April, beginning at 10 o'clock a. m., Tuesday, the 22nd day, and sitting as usual, four days.

You are hereby cordially and earnestly invited to be present and participate in the deliberations of the convention; and to lend your aid in the laudable undertaking for which this organization was instituted.

It is expected that business of unusual importance will come before this meeting; several important committees will report; and a number of papers on various subjects connected with the science of medicine have been promised. Reforms admittedly needed will be recommended to be at once inaugurated; and it is especially desirable that a full expression of the sentiment of the brotherhood of he State should be had.

Of the inducements to attend, other than the scientific interest promised by a large, and it is expected harmonious gathering of able men of the profession, it is scarcely the province of your Executive to speak; but judging from precedent, a spirit of emulation has been engendered, which will stimulate the committee of arrangements to endeavor to at least equal all past efforts in the way of entertaining our guests in a social way; the pride of the citizens will be enlisted, and it is safe to promise you on their behalf, and that of the professional brethren of that modern wonder of enterprise and public spirit, a programme, the recollection of which will ever dwell a pleasing memory with those who shall have the good fortune to be present.

The committee of arrangements will doubtless see that most favorable rates of transporatation will be secured over the several railroads centering in that city, which by the by, is alike easily accessible from all parts of the State, and is as nearly central as any point which could have been chosen.

Come! Be with us, heart and soul; and use your best influence with your professional brethren who are not members to accompany you.

With sentiments of the highest respect, I am Doctor, cordially yours, R. M. SWEARINGEN, M. D., President.

Official:

F. E. DANIEL, M. D., Secretary.

### AUSTIN DISTRICT MEDICAL SOCIETY.

Let it not be forgotten that this enterprising and progressive Society will hold its next quarterly meeting in the city of Austin, on the fourth Thursday in March (proximo). The meeting will be held in the new capitol building, which has been fitted up as a Medical Club Hall. The membership numbers nearly seventy-five, and embraces a majority of the leading physicians in the section contiguous to Austin. It is also rapidly gaining ground, and its proceedings are full of interest. Some excellent papers were read and discussed at the last meeting, which we hope to lay before our readers in due time. The programme will, as usual, be promulgated in circular form, and sent to members and others, and a large and important meeting is anticipated.

The Tri-State Sanitary Association will meet at Wheeling, W. Va., February 27 inst., and hold two days session. Dr. Harvy Reed, of Ohio, is President, and Dr. G. D. Garrison is Secretary. The object of the convention is to consider the question of floods and their results, from a sanitary standpoint, and the best methods of managing the sanitary interests of a community after such a calamity.

We regret that the announcement was received too late to be given in detail in our last issue.

THE OPHTHALMIC REVIEW begins its new volume with an American editor, Dr. Edward Jackson, of Philadelphia, who succeeds Dr. James Anderson, of London. It will hereafter contain original papers from American as well as English Ophthalmic surgeons, with a list of all papers on ophthalmological subjects, published in this country or in Europe, and full reviews of the most important of them. P. Blackiston, Son & Co., are the publishers.

---

R. RHODES REED, M. R. C. S., Norfolk, England, says: I have prescribed S. H. Kennedy's extract Pinus Chanadensis as an injection (one part to six), in an obstinate case of chronic gonorrhœa, with very satisfactory results.

The discharge considerably diminished during the first week, and after a fortnight's use the patient reported himself quite well.

